# The optoelectric tunability effect of structurally patterned Fe_3_O_4_-Au assembly on Rhodamine 6G signals under Magneto-SERS measurements

**DOI:** 10.1038/s41598-025-21536-y

**Published:** 2025-10-28

**Authors:** Paul Okpozo, Jordan C. Kelly, Jennifer A. Aitken, John Viator, Ketan Pancholi

**Affiliations:** 1 School of Engineering, Robert Gordon University, Aberdeen, AB10 7GJ UK; 2 Department of Chemistry and Biochemistry, Duquesne University, 600 Forbes Avenue, Pittsburgh, PA 15282 USA; 3 Biomedical Engineering Department, Duquesne University, 309 Libermann Hall, Pittsburgh, PA 15282 USA

**Keywords:** Magneto-plasmonics, Self-assembly, Iron-oxide, Gold, Nanoparticles, SERS, Biomedical engineering, Materials science

## Abstract

The magneto-plasmonic tunability property of magnetite-gold complex (Fe_3_O_4_-Au) colloids has garnered significant interest in bio-sensory applications like surface-enhanced Raman spectroscopy (SERS). In many studies, this tunability does not only depend on the external magnetic field contribution but also on the concentration ratio between Fe_3_O_4_ and Au. This would require multiple preparation of Fe_3_O_4_-Au colloidal badges. In this study, a magnetically stimulated Fe_3_O_4_-Au colloidal suspension in polyvinyl alcohol was spin-coated, forming a micro-patterned thin film on a silicon wafer substrate for assessing the SERS vibrational signal response of Rhodamine 6G (R6G). The varying concentration ratio between Fe_3_O_4_ and Au across three regions of interest within the single cast resulted in differing optoelectronic behaviour. Such was observed from diffuse reflectance UV-Vis-NIR spectroscopy measurements, and its impact on different Raman signals of R6G. The introduction of an external magnetic field also led to approximately 133% higher peak intensity and improved spectral resolution of R6G under SERS measurements. This implies that the magnetic field polarization of Fe_3_O_4_ domain electrons influences plasmon electrons of Au nanoparticles leading to tuning of electronic-influenced vibrations of nearby analyte molecules. The variability of nanoparticle concentration ratios, configurations and the magneto-optoelectronic effect of this design template, provide flexibility and tunability in diagnosing biomolecule signals under SERS.

**Supplementary Information:**

The online version contains supplementary material available at 10.1038/s41598-025-21536-y.

## Introduction

A label-free sensing technique like SERS involves the use of a metallic or semiconducting surface to enhance incident electromagnetic field interaction with analyte materials. This therefore leads to the amplification of their molecular vibrational signals^1–4^. The electromagnetic enhancements are made possible through the excitation of localized surface plasmon resonance (LSPR) modes in metal substrates^5^, as well as charge transfer from a semiconducting surface to molecules^6^.

The use of the Fe_3_O_4_-Au dimer system as probe nanoparticles for SERS applications has been growing in interest, where the combination of magnetite (Fe_3_O_4_) and gold (Au) nanoparticles yield advancements in optical label-free biosensory applications^7–9^. Both materials play a complementary role under the auspices of magneto-plasmonics, where the magnetic effect contributed by the Fe_3_O_4_ and the plasmon resonance effect contributed by the Au encourages its utilization in fields like biomedicine, magnetic resonance imaging and optics^10–14^. This composite is biocompatible, easily manipulatable due to its magneto-optical properties, and quite versatile in application setups, like stationary templates and dispersed colloids in flow cytometry.

Although the use of Au nanoparticles can offer enhancement factors as high as 10^7^ due to its localized plasmon resonance, Fe_3_O_4_-Au on the other hand can offer relatively higher intensities^15–19^. When considering a substrate of assembled nanoparticles for biosensory applications, the enhancement factors are influenced by the gap between probing particles influencing the lattice plasmon resonance (LPR) or surface lattice resonance (SLR)^20^. Such lattices can easily be formed using an external magnetic field to increase the degree of agglomeration of the Fe_3_O_4_-Au dimer structure, which helps by narrowing the gap between particles^18,20^. This leads to a red-shift of surface plasmon of Au and subsequently to an overall broadband absorption spectrum, that enhances the signal by as much as 14 times the factor of 10^6^^20^. This makes the micro-array setup for the SERS application attractive to several researchers, where arrays of Fe_3_O_4_-Au nanoparticles provide reproducible signals in SERS measurements^21–23^.

In another aspect of this heterostructural configuration of Fe_3_O_4_-Au, the metal-dielectric interface of these materials causes localized oscillatory charges at their boundaries upon optical excitation^24^. According to some studies, this plasmon polaritons can be manipulated by the magnetic field influenced domain polarization of the ferrimagnetic material (like Fe_3_O_4_)^25–27^. This is a quantum mechanical effect known as spin polarization of the conducting electrons. This is seen as magneto-optical effect; a way of manipulating the optical properties of a material as seen in optical switches^28,29^.

The spectra profile of this type of heterodimer structure upon UV-Vis absorption testing is influenced by the concentration ratio, particle configuration, and type of interfacial contact^30–32^. The manner of interaction causes a redshifted, reduced and broadening of the Au plasmon resonance absorption band^33,34^. This shift is attributed to electron deficiency at the Au nanoparticle surface due to its migration toward the dimer structure interface, resulting in a significant redshift of Au plasmon resonance by approximately 130 nm to 630 nm as studied by Thimsen and co^35^.

Beyond the lattice formation controlled by the magnetic field in SERS, it also serves as an effective way to manipulate the plasmon electrons through Lorentz force in the intermediate state of the Raman process, which results in the manipulation of the Raman molecular vibration intensities^36,37^. Several studies have shown changes in Raman intensity of materials using this method^38–41^. An example of such a phenomenon was executed by Ji and co., where a series of prepared magnetic responsive MoS_2_ monolayer, bilayer and bulk mode were subjected to a magnetic field with its direction perpendicular to its surface while subjected to light^42^. This arrangement resulted in the fine-tuning of optical intensity by the perpendicular magnetic field’s coupling with the material’s internal electronic and magnetic states; like in Polar Kerr effect^43,44^. The perpendicular arrangement of magnetic field maximizes the interaction between the light’s electromagnetic components (electric and magnetic fields) and the material’s electronic spins, which are aligned by the external magnetic field^25^. This magneto-optical effect stems from a magnetic-field-induced symmetry breaking for the electron motion in the inelastic Raman scattering process^45^.

This article advances prior research on the fabrication of magnetic patterning within a Polyvinyl alcohol (PVA) thin film using gold pickering ferrofluid emulsion^46–48^. The study examines the optoelectric properties resulting from diverse distributions of iron oxide (Fe3O4) and gold concentrations under SERS application. Using the concentric permanent magnet configuration, that provides multi-gradient patterned thin film structure^48^, optical investigation will be carried out in regions with distinct structural pattern difference in chain thickness and gaps. The thickness is related to the concentration of Fe_3_O_4_ and Au nanoparticles per chain that would take advantage of optical-matter interaction and electron charge dynamics within the interface of both materials. From the previous study^48^, majority of the chain thickness are similar, within ± 10% deviation, that is why in this study, three major spots with at least 20% differential was considered. In addition to such consideration, investigation on the impact of a perpendicularly aligned magnetic field on the optical behaviour of the patterned assembly complex. The research postulates that the varied distribution of Fe_3_O_4_ and Au concentrations and the linear array pattern in three distinct regions of interest will yield different optical responses within a single fabricated cast. Furthermore, the study anticipates that the effect of the magnetic field will influence the responses of biomolecular subjects during SERS tests. While acknowledging that this casting method may lack the precision of conventional lithography techniques, its vast range, rapid casting speed, and pronounced linear variability undeniably render it ideal for low-end SERS tests. Especially across a wide range of vibrational signal enhancements of test specimens.

## Materials and methods

Previous works explain the materials used for preparing magnetically stimulated patterned thin films^46–48^. The gold-Pickering ferrofluid emulsion in PVA was prepared using sol-gel processes. The preparation involved several steps: the reduction of gold(III) chloride to gold nanoparticles; the acidic reduction of a combined iron(II) and (III) chloride solution to magnetite nanoparticles, which were then dispersed in cis-cyclo-octene to form the ferrofluid; and the use of a surfactant capping agent around the gold nanoparticles to promote the Pickering emulsion of gold and ferrofluid during ultrasonication. Further details of this preparation and a schematic can be found in reference^46^.

For this study, a concentric magnetic configuration was applied to the spin-coated gold-Pickering ferrofluid emulsion in PVA. This method was chosen because it resulted in a higher degree of long-chain formation and a lower defect rate of 14% compared to the single permanent magnet configuration^48^.

**Fig. 1:**
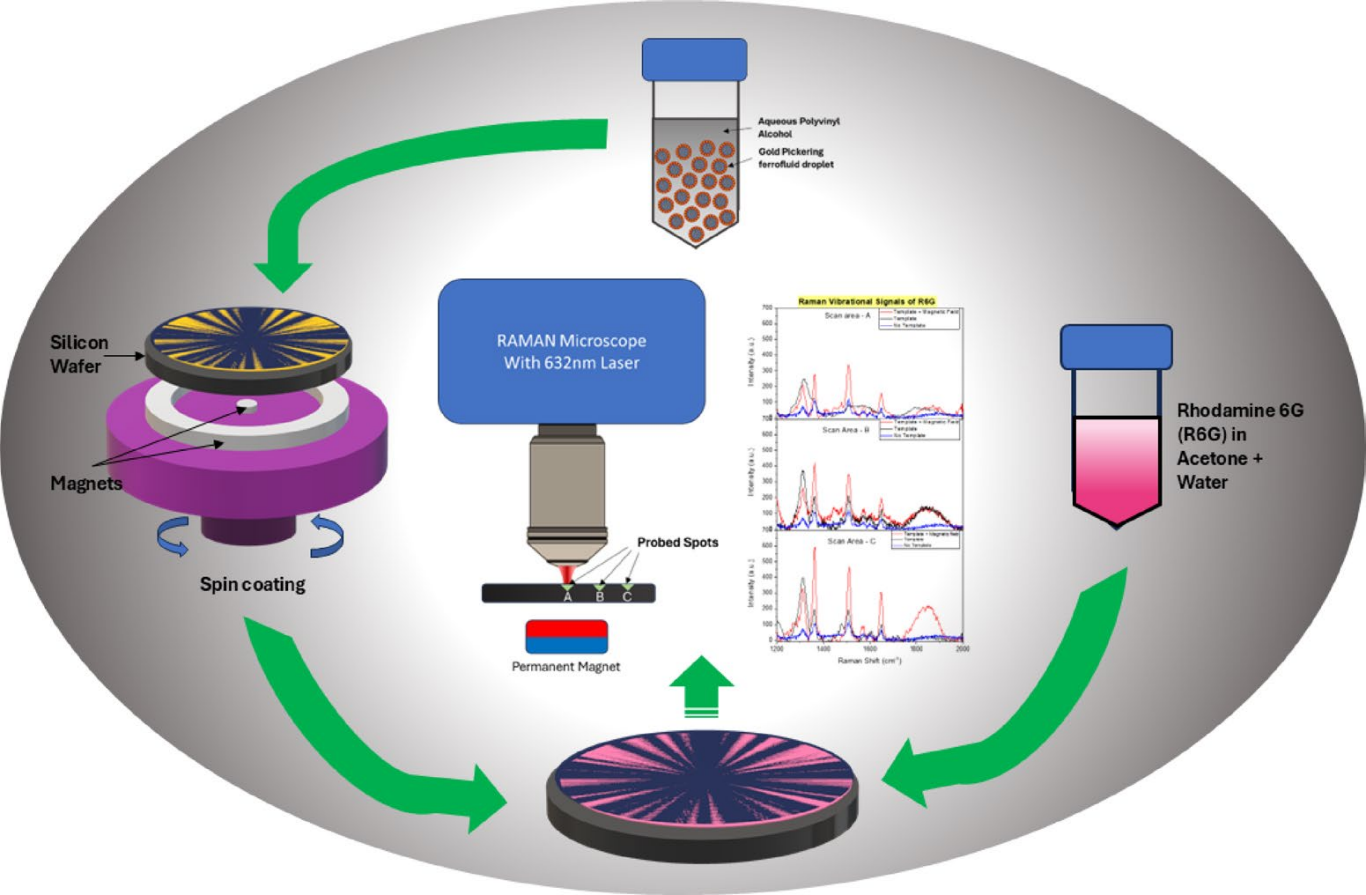
Schematic of investigation process.

The procedure for this study can be seen in the schematic shown in Fig. 1. The goal of the research went beyond simply preparing a patterned thin film; it was to understand the optical contribution of this pattern using Raman spectroscopy. This was to be done by analysing three distinct regions with at least a 40% difference in their chain thickness or chain gap ratio, ensuring that the signal responses could be easily differentiated.

The decision to use Rhodamine 6G (R6G) as the probe molecule for Surface-Enhanced Raman Scattering (SERS) was based on its well-documented multicomponent bonds and Raman vibrations, as corroborated in previous studies^49,50^. The use of R6G can help identify these differences through its multiple fingerprint Raman shift bands.

Furthermore, we will investigate the provision of a perpendicular magnetic field to the thin film on the substrate during the Raman test. This will be achieved by applying a single permanent magnet beneath the substrate to observe its contribution to the optical performance of the patterned structure.

### Materials

Preparatory materials for the production of gold-pickering ferrofluid emulsion can be found in previously published works^46–48^ (see also TEM micrograph in Figure 3(f)). SCS™ 6800 spin coater, 25 mm OD, 15 mm ID and 5 mm thick ring-type, and 3 mm diameter, 2 mm thick cylindrical Neodymium Magnets purchased from First4magnets^®^ UK. Also purchased was 50 mm diameter by 3 mm thick Neodymium disc magnet N52 from Magnetstore UK. VTSYIQI^®^ probe type gaussmeter with measurement scope from 0–200mT (1mT = 10Gs), resolution of 0.1mT. Rhodamine 6G (MW 479.01 g/mol) and Acetone (ACS reagent, ≥ 99.5%) were purchased from Sigma Aldrich. Silicon wafer was purchased from Pi-Kem, and it possesses properties such as; *crystal growth*-float zone, *grade* - prime or optical, *diameter* – 4”, *type* - p-type, *dopant* - boron, *orientation* − 100, *resistivity.*

### Methods of Preparation

**Fig. 2:**
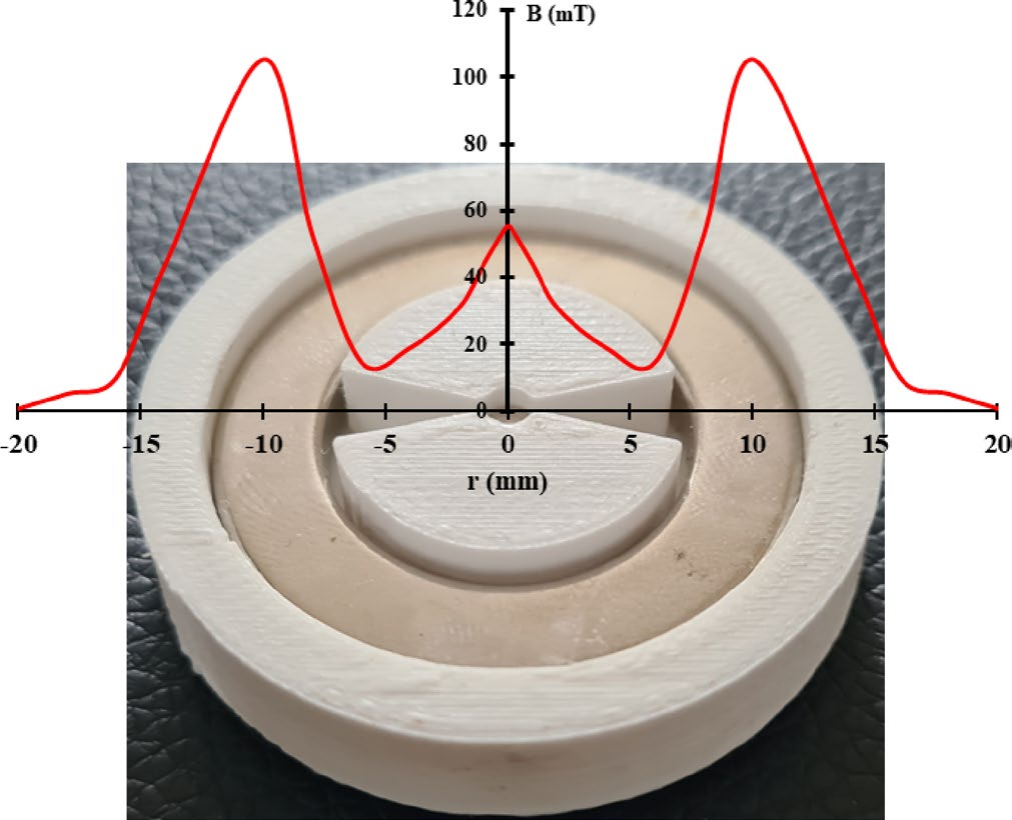
Neodymium permanent magnetic setup in the 3D PLA housing and the measured magnetic field strength distribution (mT) across the diameter (mm) of the setup.

The method of preparation of Au-Pickering emulsion in PVA and Au-Fe_3_O_4_ patterned thin film is explained in previous work^48^. The particular configuration is a concentric magnetic setup shown in Fig. 2. The magnetic housing was 3D printed with PLA, and it weighed 0.96 g.

The nanopatterned thin film was prepared using gold-pickering ferrofluid emulsion in PVA of 15 mPas viscosity and at spin coating speed of 3000 rpm for 60 s. This condition dries the aqueous solution into a thin patterned film. This film on the silicon wafer was studied using UV-Vis-NIR spectroscopy.

For the test sample in the Raman studies, approximately 4.6 × 10^−7^M of R6G in 95% acetone and 5% DI water was prepared, and the thin-film nanostructured coated wafer was immersed and left for 5 h. Acetone was used because it does not react with PVA and provides enough time to bind R6G to the nanostructured thin film (15.2 mPas PVA, 3000 rpm spin speed).

### Diagnostic equipment and procedure

#### Optical microscopy and image analysis

Optical images of samples were captured using an Olympus^®^ BX41 Darkfield microscope attached with M-plan Achromat MPLN-BD 50X NA 0.5. All 1920 × 1080 pixel images were obtained with a resolution of 0.17 μm/pixel. The images were analysed using the Image J^®^ tool, where sections of the thin film were converted to binary format, causing the pickering droplets to appear white on a black background. The contrast between the two allowed for the identification of the number of white pixels in a unit scan profile of interest representing the chain thickness and gaps using the image analysis^46,47^.

#### Vibrating sample magnetometer and magnetic hysteresis curve

Three 5 mm square area of coated silicon wafer hosting the interested regions of the thin film (PVA viscosity of 15.3mPas, spin speed of 3000 rpm) with approximate density of 2421 Kg/m^3^ was tested using the Vibrating Sample Magnetometry (MPMS, Quantum Design, Inc) at 300 K. This was to estimate the coercivity, saturation magnetization, and susceptibilities of the iron oxide (Fe3O4) patterned nanoparticles across the regions of interest.

#### Grazing incidence X-ray diffraction of Fe_3_O_4_-Au thin film

A Malvern Panalytical Empyrean 3 multipurpose X-ray diffractometer with an X’cellerator detector operating in Bragg-Brentano geometry with a Cu-K_α_ radiation (λ = 1.541871 Å) was used for data collection. The samples were mounted on a multi-axis cradle or stage, allowing for precise control of the sample’s orientation and position relative to the incident X-ray beam. The thin film was analyzed through grazing incidence X-ray diffraction using a flat sample stage with a fixed omega of 0.5° to limit diffraction by the silicon wafer. Data were collected from 10 to 80° 2θ with a step size of ∼0.0084° and scan speed of ∼0.0048° s^−1^. The incident beam optics included a 0.04 rad Soller slit, an automatic programmable divergent slit, and a fixed 2° anti-scatter slit, while the diffracted beam optics consisted of a 0.04 rad. Soller slit, automatic programmable anti-scatter slit, nickel beta filter, and a scanning line detector. Data processing and phase identification were carried out using the X’Pert Highscore Plus software with the powder diffraction file (PDF) database from the International Centre for Diffraction Data (ICDD). The sample was positioned to observe diffraction at three different locations on the thin film (A, B, C) to analyze the concentrations of Fe_3_O_4_ and Au qualitatively.

#### Optical diffuse reflectance UV-Vis-NIR spectroscopy

Optical diffuse reflectance UV-Vis-NIR spectroscopy data were collected using a Cary 5000 UV–vis–NIR spectrometer with BaSO_4_ (99.92%, Fisher Scientific) being used as a 100% reflectance standard^51^. A 3D printed support was made to support the coated substrate to match the optical scan spot for optimal reflectivity at 10^o^. The scans were performed from 200 to 2500 nm at a rate of 600 nm/min. The percent reflectance data were converted to absorption using the Kubelka–Munk formula^52^. The 50 mm diameter by 3 mm thick N52 magnet was placed directly underneath a different 3D printed support for the coated substrate matching the primary position and orientation of the sample. Before commencing test, the magnetic field distribution across the surface of the sample was checked using the VTSYIQI^®^ probe type gaussmeter, and the average field strength across the regions of interest was approximately 200mT.

#### Raman microscopy

Renishaw in ViaTM confocal Raman microscope, using a 2 × 10^3^ W/cm^2^ powered 633 nm laser Renishaw CCD Camera detector, and a 50X, NA 0.55 objective lens. Data processing is standard, such as offset and cosmic ray removal. Upon the arrival of data, baseline correction via Origin ^®^ was undertaken, and the vibrational signal intensities were examined and relatively compared.

In the first step of investigation, an uncoated silicon wafer was tested under varying laser power (0.1%, 1%, 10% and 100%) by adjusting the neutral density filter (Supplementary information Figure S-4a). The second step involved testing the substrate coated with the thin film structure with a 0.1% incident laser power to observe the enhancement contributed by the patterned nanoparticles across regions A, B and C (Supplementary information Figure S-4b). During these stages of diagnosis, 520 cm^−1^ peak was taken as the point of reference. The purpose for these test is to be used as a comparative reference for the laser intensity selection.

The third step involved testing R6G coated on bare silicon wafer, and then on the patterened thin film across the three locations of interest (A, B, and C). The final step involved the same procedure in the presence of a 200 mT magnetic field across these regions of the substrate (see Fig. 1 schematic), and output in (Supplementary information Figure S-4c).

#### Scanning electron microscopy (SEM)

A 500 °C calcinated spin-coated sample was tested using a Quanta 650 FEG SEM. Depending on the magnification, a working distance of 10 ± 1 mm was used for the scan, along with a low-pressure vacuum of 0.825 Torr and an accelerating voltage for electrons (HV) of 20 kV. The purpose of the SEM was to examine areas with high resolution of the patterned structure closer at higher magnification.

## Results

**Fig. 3:**
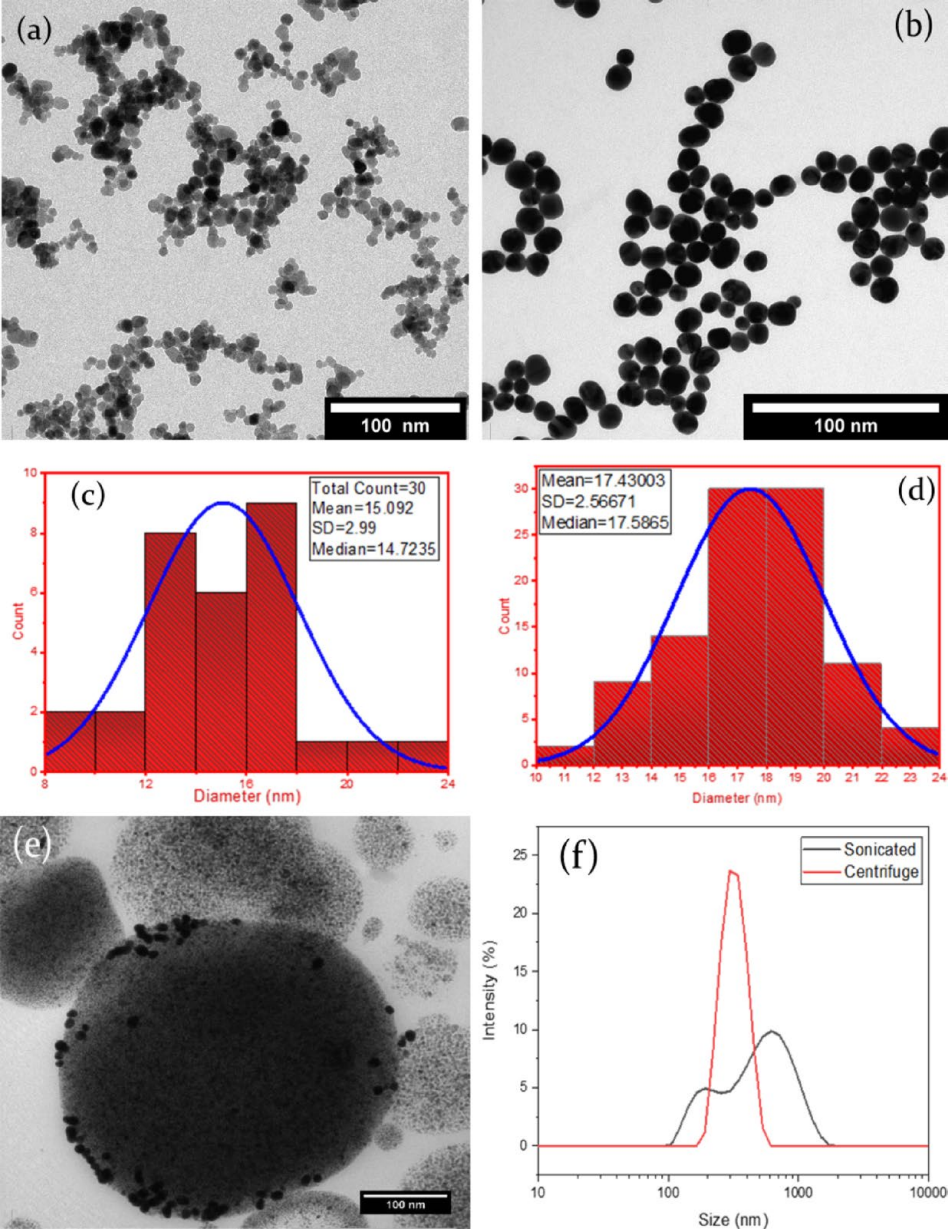
TEM images of: **(a)** Fe_3_O_4_ and **(b)** gold with their respective particle size distribution histogram (**c** & **d**), **(e)** Au pickering ferrofluid droplet **(e)** TEM images of Au pickering ferrofluid droplet. **(f)** Size distribution of ultrasonicated Au pickering ferrofluid emulsion averaging 600 ± 500 nm (Black line), and after centrifugation with distribution averaging 330 ± 200 nm. (**a**, **b**, **c**, **d** & **f**) are reproduced from Ref. 47 with permission from Hybrid adv., Elsevier, **(e)** is reproduced from Ref 46 with permission from the Royal Society of Chemistry).

The outcome from the preparation of magnetite and gold nanoparticles was observed using TEM (Fig. 3a and b respectively) and their size distribution was quantified using image J processing software. The magnetite (Fe3O4) nanoparticles averaged approximately 17 nm, while gold was 15 nm (Fig. 3c and d respectively). The TEM image shown in Fig. 3 (e) is a dried sample of gold-pickering ferrofluid droplet under ambient condition; with the black Au nanoparticles (referencing Fig. 3b) circumferentially distributed about the bead of densely agglomerated Fe_3_O_4_ nanoparticles; with several Au nanoparticles dislodged due to evaporated/drained cis-cyclooctene^34^.

Figure 3 (f) represents the bimodal size distribution of ultrasonicated gold pickering ferrofluid emulsion averaging 600 ± 500 nm. Then with size management approach using centrifugation, spun at 4500 rpm for 10 min yielded modal distribution averaging 330 ± 200 nm^35^.

**Fig. 4:**
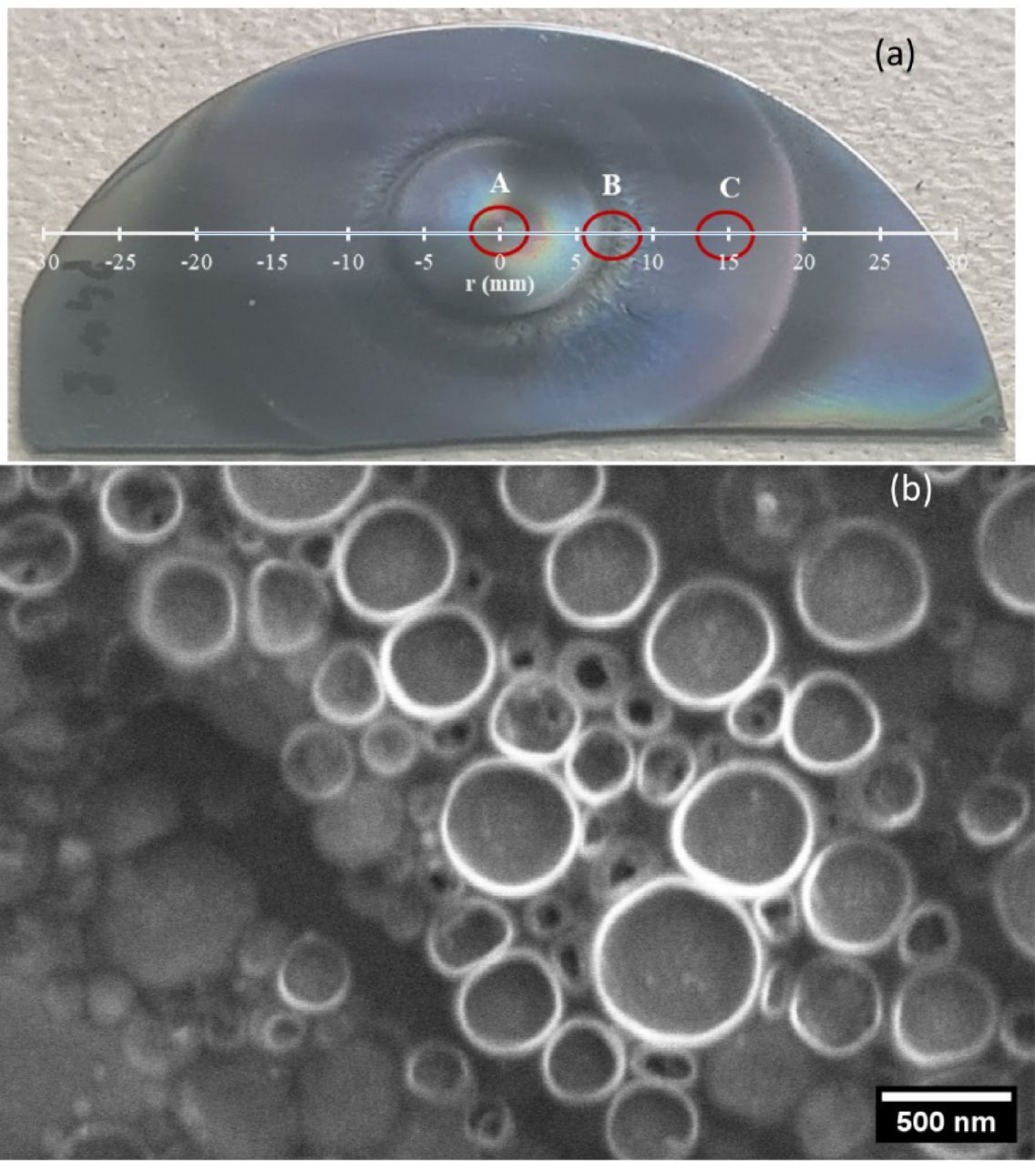
**(a)** Magnetically patterned Au-pickering-ferrofluid emulsion in PVA thin film after spin coating at 3000 rpm on a silicon wafer. A, B, and C are spectroscopically scanned locations in the thin film. **(b)** SEM image of 500 °C calcinated sample taken at region A, with Fe_3_O_4_ as the dark circular structure and Au (brighter nanoparticle) distribution.

Figure 4**(a)** shows the spin-coated sample of the composite material on a silicon wafer. The color gradient from the middle indicates optical birefringence controlled by the degree of gold-pickering-ferrofluid chain clusters. To examine the structure formation more closely, the sample was calcinated in an oven at 500 °C for 1 h to remove the PVA film. The SEM microcrograph taken at region A of the sample shows oval-shaped structure of magnetically agglomerated ferrofluid droplets containing mainly Fe_3_O_4_ nanoparticles **(**Fig. 4b**)**.

The rest SEM micrographs for regions B and C can be found in supplementary information (Figures S-1 to 3).

### Optical microscopy and image analysis

**Fig. 5:**
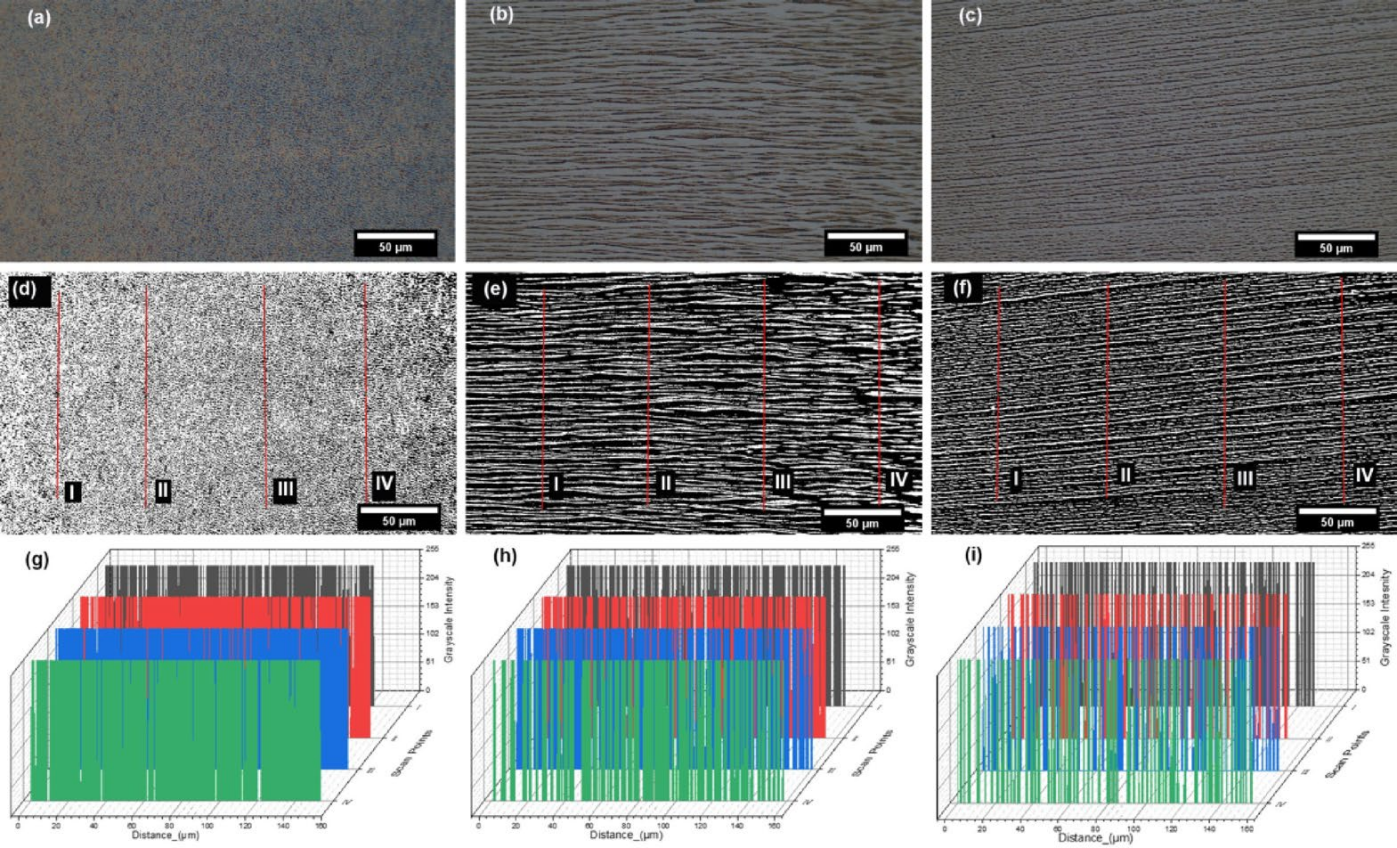
**(a-c)** Darkfield microscopic images of positions A, B & C, respectively. **(d-f)** binary processed images of respective photos A, B & C, with red lines for plot profiles for identifying chain thickness and chain gaps. **(g-i)** are the respective A, B & C plot profiles of scanned lines (I, II, III & IV) covering the distribution of chain thickness and gap.

Microscopy images were taken to observe and measure the grating pattern morphology across three (A, B and C) regions of the thin film. Figure 5 (a-c) shows the darkfield microscopy images of the three regions accordingly. Samples of the magnetic stimulated packing of gold pickering ferrofluid droplets was captured from the three regions using SEM, and they are shown in supplementary information S.1 (Figures S-1 to S-3). In order to create a profile distinction between droplet-packed chains and the space between them (gaps), the imageJ processed binary images that contain red lines (I-IV) (Fig. 5 (d-f)) was employed for estimating the average chain thickness (white at 255 grayscale) and gaps (black at 0 grayscale). Analysing these optical images for chain thickness and gap was because of it’s larger field of view (approx. 60,000 µm^2^) and range of distribution covered; which approximately matches the field of view by the incidence of Raman laser on the samples. With the resolution of these images being 168 nm/pixel, the data of each scan profile line were plotted on a 3-dimensional graph (Fig. 5g-i). The images and respective profile plots show the degree of packing density of Pickering droplets in each region, with ‘A’ having the highest and ‘C’ the least, with a growing degree of gaps. The data from the plot profiles were further processed using a designed algorithm (Supplementary S.2) that helped in quantifying average chain thickness and gaps with their respective standard deviations. Table 1 shows a summary of the final quantified values of each scanned region A, B and C. CntCT and CntCG are the average pixel counts of chain thickness (CT) and chain gap (CG), respectively, for each scanned points I, II, III & IV.

The data from this image would represent the droplet packing density by chain thickness that would be related with the optical behaviour of the sample. Noting that more particles would amount to more optical-matter interactions.

It can be seen from the quantification of CnCT the decrement from region A to C, while CntGap increases (Table 1). This is as a result of the combined effect of counteracting forces in magnetism and fluid inertia from centrifugal action as previously investigated^48^.

**Table 1:** Summary of quantified binary processed images of regions A, B, and C, with CntCT and CntCG as pixel count per chain thickness (CT) and gaps (CG), respectively, and SD as standard deviation.

		Scanned Points											
		I		II		III		IV		**Average (I**,** II**,** III**,** IV)**		**Standard Deviation**	
**Region**	**A**	CntCT	CntGap	CntCT	CntGap	CntCT	CntGap	CntCT	CntGap	CntCT	CntGap	SD CT	SD CG
	Pixels (avg)	13.93	2.27	13.55	1.75	12.7	1.15	12.12	1.67	13.075	1.71	0.82	0.46
	*pixels X 168 [nm/pixel]*									***2197 [nm]***	***287 [nm]***	***137 [nm]***	***77 [nm]***
	**B**	CntCT	CntGap	CntCT	CntGap	CntCT	CntGap	CntCT	CntGap	CntCT	CntGap	SD CT	SD CG
	Pixels (avg)	9.65	5.79	9.73	5.23	10.06	6.61	8.54	7.16	9.495	6.1975	0.66	0.86
	*pixels X 168 [nm/pixel]*									***1595 [nm]***	***1041 [nm]***	***111 [nm]***	***143 [nm]***
	**C**	CntCT	CntGap	CntCT	CntGap	CntCT	CntGap	CntCT	CntGap	CntCT	CntGap	SD CT	SD CG
	Pixels (avg)	7.73	9.27	7.02	9.92	7.37	9.71	7.27	9.75	7.3475	9.6625	0.29	0.28
	*pixels X 168 [nm/pixel]*									***1234 [nm]***	***1623 [nm]***	***50 [nm]***	***47 [nm]***

By observing CnCT across the regions (A, B and C), it was realized that each consecutive region (A and B) and (B and C) differed by 27% and 23% respectively; thus above the set targeted difference of 20%.

### Magnetization curve

**Fig. 6:**
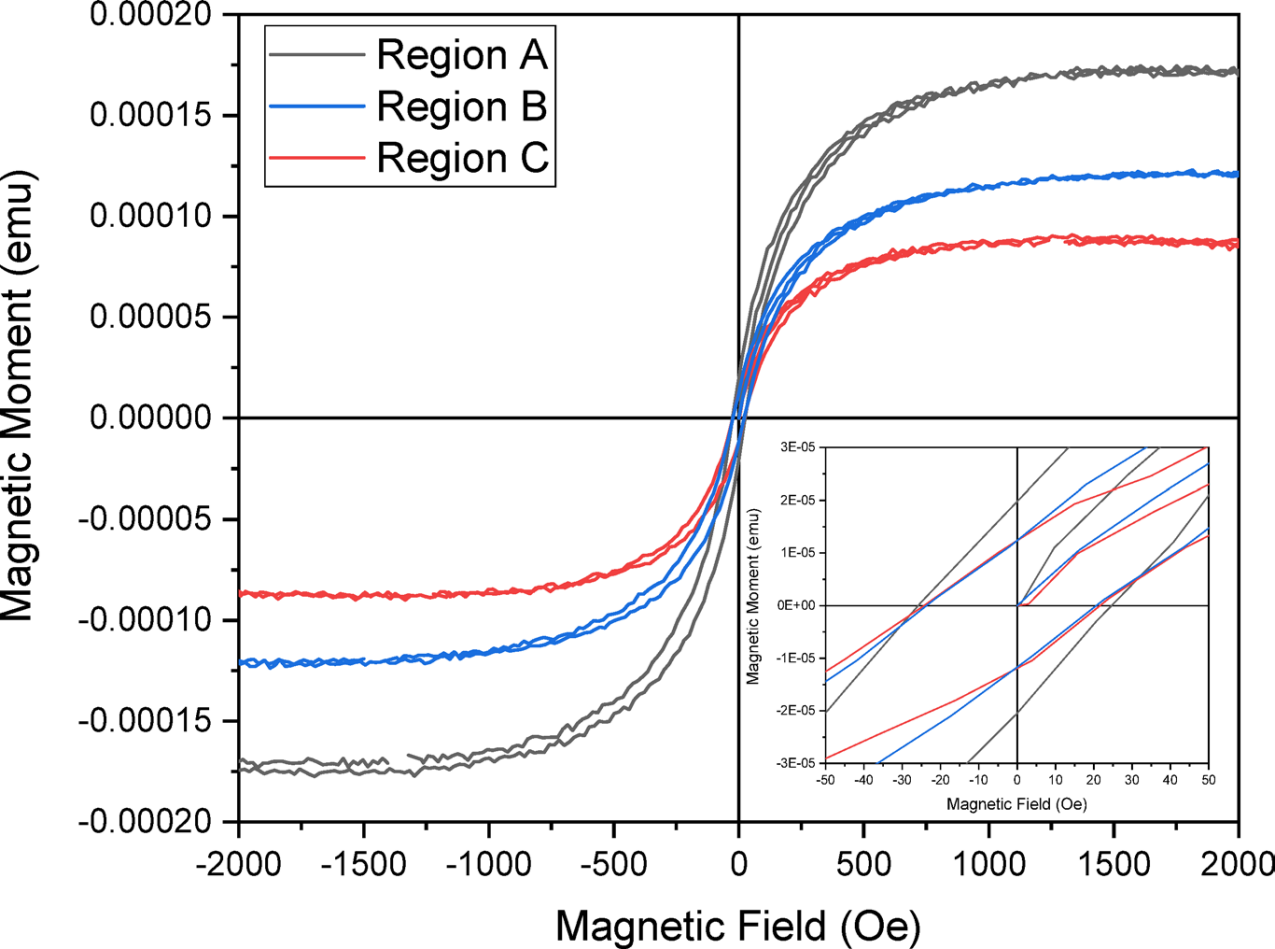
Magnetic Hysteresis curve of 5 mm cut of coated sample on the silicon wafer. Insert is a magnification of magnetization curve at the origin for estimating the coercivity and magnetic susceptibility.

The hysteresis loops of patterned thin film coated on a silicon wafer is shown in Fig. 6. The insert in the figure shows a zoom-in at the curve intersecting at both sides of the axes. The units were converted to determine the necessary parameters. 1 kA/m = 1(emu/g X density of tested sample (g/cm^3^)), 1 Oe = 79.56 A/m.

From the calculations, the coercivity (H = 0) for nanoparticles in regions A, B, and C are 2.23 kA/m, 1.99 kA/m, and 1.91 kA/m, respectively, and the magnetic saturations are 0.43 A/m, 0.3 A/m, and 0.21 A/m, respectively.

**Fig. 7:**
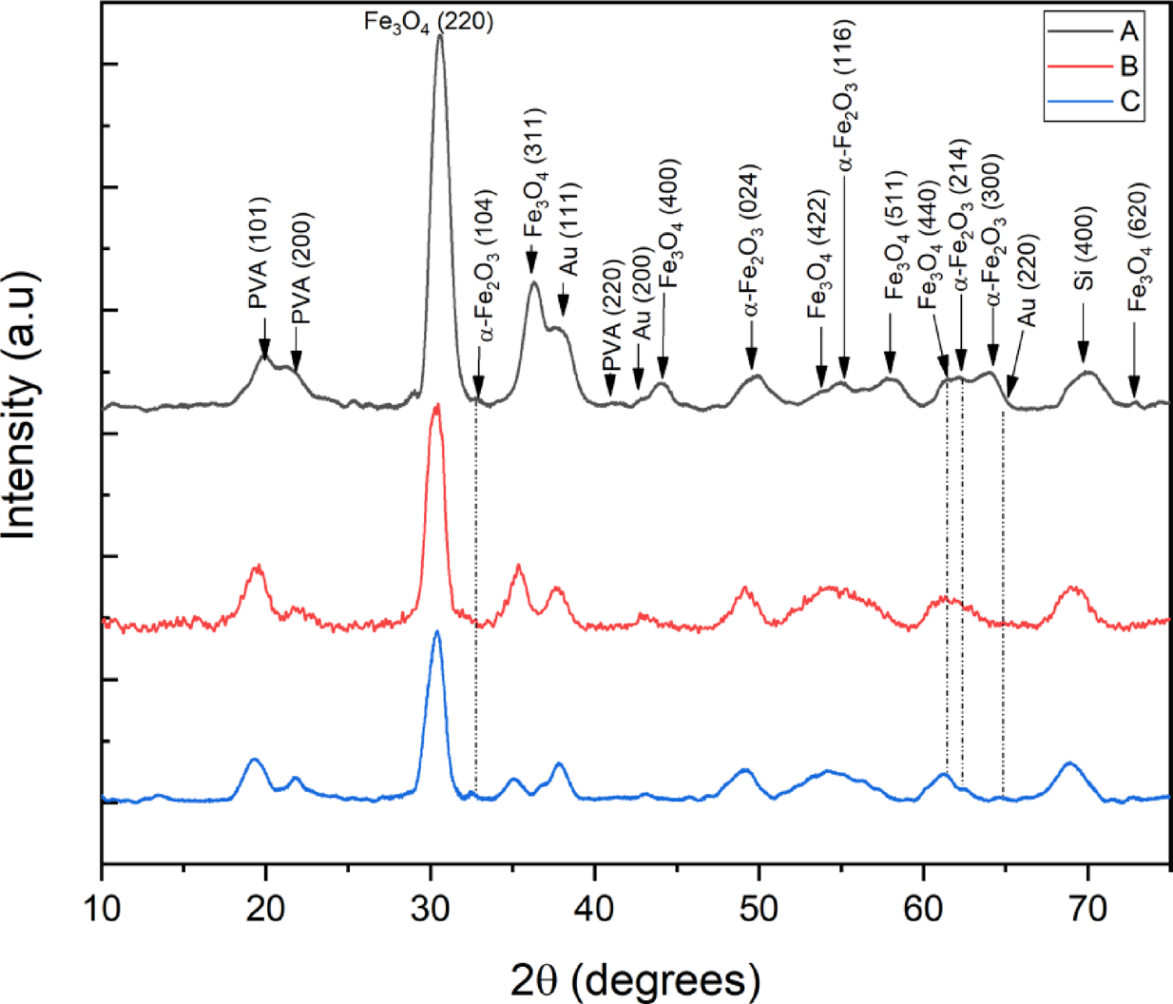
Grazing incidence X-ray diffraction patterns collected on a 3-layer thick Fe_3_O_4_ + Au thin film at positions A (black), B (red), and C (blue).

The coercivity plays an important role in ferromagnetic materials that possess multi-domains with unequal magnetization in the bulk form^53^. Beyond that size, particle has more than a single domain and coercivity reduces due to energy consumed for domain wall formation; thus would require more field strength to get magnetized up to saturation levels. From a previous study involved with testing the magnetic property of Fe_3_O_4_ nanoparticles, a coercivity of 1.275 kA/m was realised^47^. Compared to dried powder of Fe_3_O_4_ nanoparticles tested, the nanoparticles within the ferrofluid droplet had the room of rotation due to magnetic torque exerted by their magnetic moment to align within its most stable orientation during magnetization prior to spin coating^54^. After drying, these Fe_3_O_4_ nanoparticles were captured or frozen within the chain domain. Therefore upon testing, it was observed that the coercivity values across the three regions were higher than the coercivity of the synthesized magnetite powder. Chain-oriented magnetite particles generally exhibit a higher coercivity compared to dispersed magnetite nanoparticles. This difference arose from the strong magnetic interactions within the chain structure, which made it more difficult to reverse the magnetization^55,56^. The orientation of the particles within the chain caused the magnetic moments to align along the chain axis, further strengthening the magnetic interactions. Obviously, because of the degree of agglomeration across each region; region A showed to be the highest because of its higher degree of dense chain networks (Fig. 7; Table 1), that provided higher degree of magnetization, thus the highest in magnetic saturation value compared to the other regions.

### Grazing incidence X-ray diffraction of Fe_3_O_4_-Au thin film

Grazing incidence X-ray diffraction was used to identify material components at the different positions on the sample (Fig. 7). In specificity, because the thin film sample is circular in formation, a symmetry was set across the middle to analyze opposite portions of the same sample at their respective measured spots A, B and C. Overall, three samples were tested and the spectra was smoothed using Savitzky-Golay filtering with 2nd degree order of polynomial regression to the data points via Origin software^57^. A fixed omega of 0.5° led to a minimal signal from the Si substrate so that other materials can be detected and distinguished. From the scans, PVA and Au were detected, as well as a polymorphic heterostructured iron oxide material in magnetite (Fe_3_O_4_) and hematite (α-Fe_2_O_3_). The existence of this iron oxide heterostructure was a result of the sol-gel co-precipitation technique used for the preparation of Fe_3_O_4_, where interfacial parts of the nanoparticle were partially oxidised^58–60^.

Grazing incidence X-ray diffraction was mainly used to qualitatively assess the proportions of Fe_3_O_4_ and Au at three locations on the thin film (Fig. 7). The patterns shows peaks at 2θ = 30.2, 35.6, 43.2, 53.7, 57.2, and 62.8, which are assigned to the reflections of the planes of Fe_3_O_4_ (220, 311, 400, 422, 511 & 440 respectively), while peaks at 2θ = 38.2, 44.4, and 64.7 are assigned to reflections of the (111), (200), and (220) planes of Au, respectively, in all probed locations (A, B and C)^61,62^. The peaks corresponding to Fe_3_O_4_ (311) and Au (111) at ~ 35° and ~ 38° 2θ, respectively, are selected to represent the relative concentrations of each component deposited on the substrate; a technique utilized in a different study^63^. Position A possesses a higher concentration of Fe_3_O_4_. At the same time, Au is found in larger amounts at position C. The results support the data obtained through the diffuse reflectance UV-Vis-NIR spectroscopy experiments.

### Diffuse reflectance UV-Vis-NIR

**Fig. 8:**
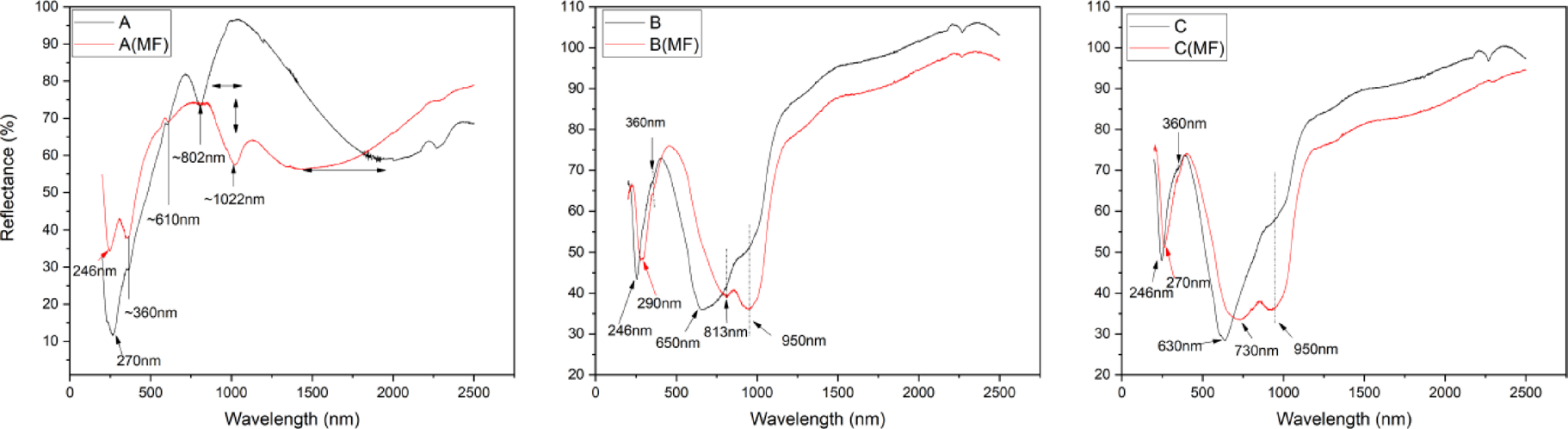
UV-Vis-NIR diffuse reflectance plot of magnetically patterned thin film with and without 200mT magnetic field (MF) across probed regions **(a)** A, **(b)** B, and **(c)** C.

The UV-Vis-NIR reflectance data of scanned spots A, B and C shown in Fig. 8 consists of readings *with* (red) and *without* (black) magnets placed. In Fig. 6, the scanned “A” region *without* the magnet shows a spectra band between 240 nm and 360 nm with a peak at 270 nm. This can easily be assigned to the intrinsic band gap absorption of the magnetite nanoparticles^64^.

Au nanoparticles under electromagnetic radiation exhibit localized surface plasmon resonance (LSPR) in the visible range (~ 520 nm for spherical NPs), driven by coherent electron oscillations under light excitation^65,66^. Fe₃O₄ on the other hand is a high dielectric medium that modifies the electromagnetic environment near Au NPs via dielectric screening. Generally, in the range of 500 nm to 600 nm wavelength, a visible peak/dip in reflectance can be seen which indicates shifting and broadening changes in the local dielectric environment due to Fe_3_O_4_ coupling. While spectra beyond 1000 nm reflects polaronic conductivity; in other words small bandgap semi-conductor behaviour of Fe_3_O_4_^67^.

In region A (Fig. 8a), under no magnetic field, a single absorption band at 270 nm is observed, primarily attributed to the sol-gel prepared Fe₃O₄ nanoparticles. The strong absorption in the UV-Vis range is largely due to electronic transitions, particularly charge transfer transitions (e.g., O²⁻ → Fe³⁺ and intervalence charge transfer between Fe²⁺ and Fe³⁺)^40,68–70^. Band at 610 nm characterizes a normal Au and Fe₃O₄ interaction via weak electromagnetic coupling or dielectric screening^71,72^. Meanwhile at 802 nm a hybrid (or a complex) plasmon mode exist between weakly coupled Au lattice surface plasmon resonance (LSPR) and Fe₃O₄ strong aggregation^26,73^. At the region of ~ 2000 nm Fe₃O₄ exhibits intrinsic optical absorption/reflectance features that supports broad near infrared (NIR) absorption^74–76^. This is influenced by its electronic band structure, mixed valence states, and potentially free carrier absorption^25^. The broadness could be due to the inherent random magnetic domains and disordered spins in the absence of an external field^25,26^. Then because of the ferrofluid droplet (~ 200 ± 50 nm in diameter) hosting agglomerates of Fe₃O₄ nanoparticles, there are retardation effects that shifts the plasmon to longer wavelengths.

Under static magnetic field, the 270 nm band appears to evolve, leading to two prominent peaks at 246 nm and 360 nm. 246 nm absorption peak potentially represents intervalence charge transfer between Fe²⁺ and Fe³⁺ of Fe₃O₄^77^. The emergence of the 360 nm peak, along with the spectral evolution, is indicative of a strong magneto-optical effect within the dense Au-Fe₃O₄ clusters. The 610 nm peak is unaffected because Au LSPR is non-magnetic^71^. The 802 nm peak on the other hand was suppressed because the external magnetic field decoupled the weak Au-Fe₃O₄ hybrid modes by aligning Fe₃O₄ spins^26,71^. The opposite happened for the 1022 nm band which is a characteristic of its semiconducting/semimetallic nature and electronic transitions within the iron ions (Fe²⁺ and Fe³⁺) in its crystal structure^43,44^. As for the broad reflectance band centered at ~ 2000 nm, there was a significant blue shift to 1400 nm due to the parallel magnetic moment alignment of Fe₃O₄ domains potentially inducing changes in its electronic band structure, charge carrier dynamics, or suppressed scattering losses^44,74,75,78^.

In region B (Fig. 8b), 246 nm peak potentially represent the electronic charge transfer transitions (e.g., O²⁻ → Fe³⁺ and intervalence charge transfer between Fe²⁺ and Fe³⁺) of Fe₃O₄^44,68,77^. The 650 nm peak is about the weak coupling of Au to Fe₃O₄ that created a red-sfift of Au LSPR under the Fröhlich condition^24,79^. The 950 nm band peak involves the multi-domain lossy magnetic plasmon mode localised at the Au-Fe₃O₄ interface, where Fe₃O₄ creating low energy with disordered spins and Au acting as a passive enhancer of scattering efficiency (radiative decay) via near-field coupling; similar to the 1022 nm in region A. The slight shift from 360 nm to 390 nm under the external magnetic field​ induces charge transfer at the Au-Fe₃O₄ interface. Fe₃O₄’s spins align, creating a local magnetic moment that polarizes Au’s conduction electrons. This changes the dielectric environment around Au. Also the band between 240 nm and 290 nm weakens because the magnetic field suppresses spin-flip interband transitions in Fe₃O₄; since UV absorption is dependent on this spin flips of Fe²⁺/Fe³⁺ mixed valence state. This is evidenced in literature on UV suppression of magnetized Fe₃O₄ thin films^80,81^. Magnetization initiated a reduction in the 650 nm plasmon band of Au within the Fe₃O₄ dielectric environment that consisted of both longitudinal and transverse (transverse dominant), and then polarizing the plasmon resonance to a strong 813 nm band. This band is simply the longitudinal magneto-plasmon mode that emerges when the applied field makes the Au electrons oscillate along the chain axis in a gyrotropic Fe₃O₄ environment^25,71^. The increased intensity of 950 nm band upon sample magnetization emanates from Fe₃O₄ spin precession leading to damping at interfaces of Au-Fe₃O₄ which contributes to ~ 75% of light energy absorbed, especially when Au acts as a field concentrator that enhances this magneto-optic loss.

The UV-vis-NIR spectra for region C (Fig. 8c) is similar to that of region B, where 630 nm is from the coupled hybrid plasmon from Au-Fe₃O₄ interfaces. Under magnetic field, the Fe₃O₄ spins align spin-orbit interaction which contributes to the spin-mediated decoupling at the Au-Fe₃O₄ interfaces reducing the 650 nm plasmon band into field-induced anti-bonding hybrid plasmon at 730 nm (a higher energy) and 950 nm (a lower energy state)^25,82^.

Overall, this system demonstrates an interesting magnetoplasmonic behaviour via magnetoelectric coupling from the magnetically aligned domain of Fe3O4 nanoparticles establishing a dielectric modulated influence on plasmonic charged electrons; with region A providing drastic magnetoplasmonic effect, and regions B and C providing finer and more sensitive modulation.

### Raman

Prioir to the main test involving the prepared sample with R6G, Raman test was carried out on patterend thin film on silicon wafer to observe the vibrational intensity effect under 0.1% of incident laser power at the three region of interest and under the application of 200mT magnetic field. More details of the test can be found in the supplementary information (S. 3).

**Fig. 9:**
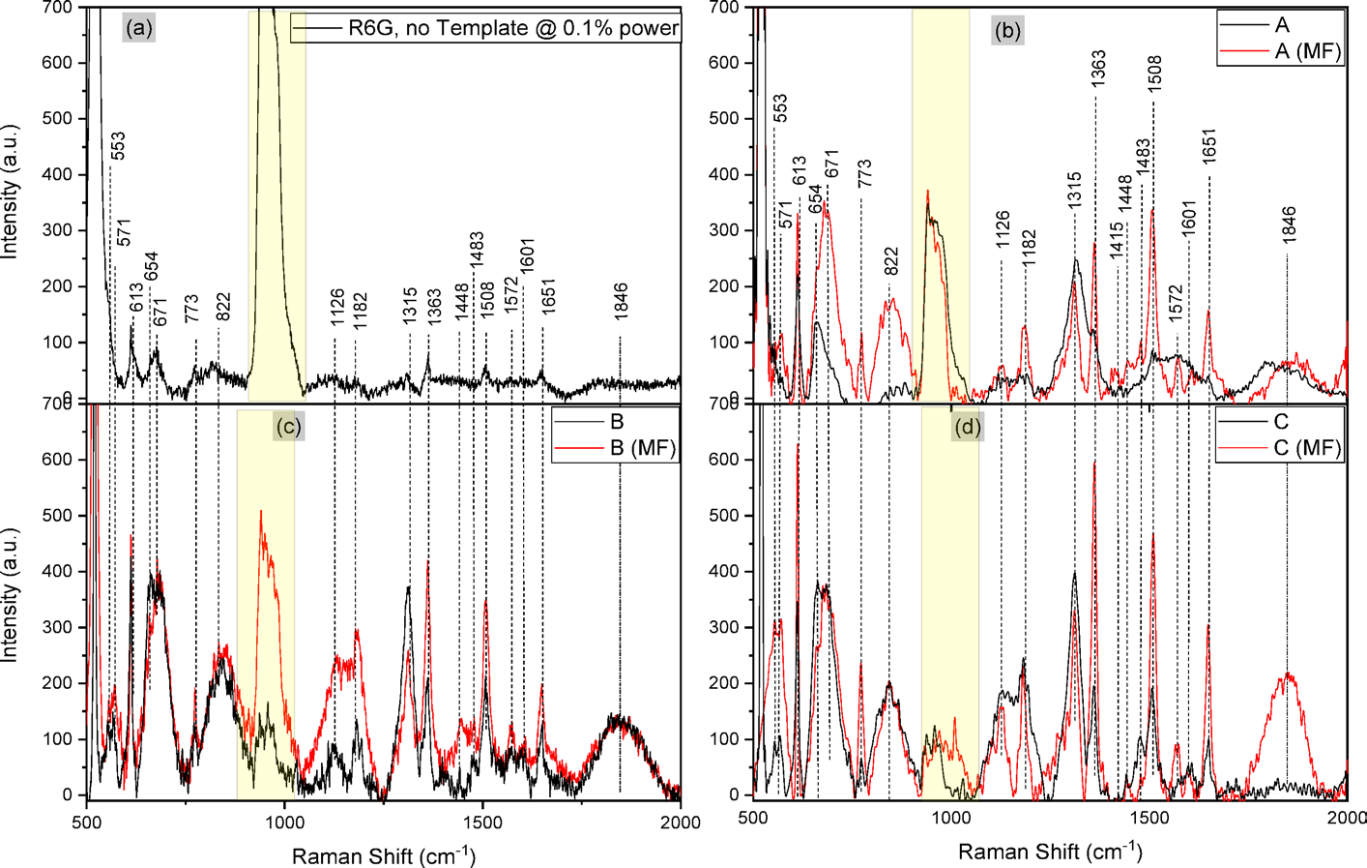
**(a)** R6G on PVA-coated silicon wafer (SW) subjected to 0.1% of 633 nm edge laser power. R6G Raman shifts at locations A **(b)**, B **(c)** and C **(d)** with and without 200mT magnetic field (MF). The yellow zone represents the silicon wafer band.

The Raman spectroscopy test for R6G was firstly carried out, dried over a bare silicon wafer substrate, and measured under 0.1% of illumination power for the purpose of effective comparison with that of the patterned thin film template (Fig. 9). The average peak intensity of R6G with no template (Fig. 9 (a)) hovers about 60 (a.u.) of the recorded vibrational intensity value, along with the indicated Raman shift bands and each necessary assignment (Table 2).

The raw data was processed by initiating baseline correction in order to observe and evaluate the peak intensities of the raman spectra. This is because local enhancements are realized in the vibrational modes of the probed specimens^83^.

**Table 2:** Identified Raman bands of R6G, with assignments referenced from^84–86^.

Raman Shift [cm^−1^]	1651	1601	1572	1508	1483	1448	1363	1315
Assignment	Aromatic C-C stretching in Xanthene ring	Hybrid mode in Phenyl ring with COOC_2_H_5_	Aromatic C-C stretching phenyl ring	C-C stretching in Xanthene ring	Antisymmetric strecthing vibration mode of CO_2_^−3^ ions	C-N stretching in NHC_2_H_5_	Aromatic stretching in Xanthene ring	Aromatic C-C stretching
Raman Shift [cm^−1^]	1182	1126	940	773	671	611–613	571	553
Assignment	C-H in plane bending in Xanthene ring	C-H plane breathing in Xanthene/Phenyl ring	Ring Breathing	C-H out of phase bending	C-C-C ring in plane bending in Xanthene/Phenyl rings	C-C-C ring in plane bending in Xanthene/Phenyl rings	Associated with Si	Associated with Si

The highlighted yellow region is associated with the silicon of the substrate. At region A without magnetic field (Fig. 9(b)), the Raman signal of R6G appears significantly higher than the outcome of Fig. 9(a), particularly at 613 cm^−1^ (75%), 1126 cm^−1^ (60%), 1315 cm^−1^ (67%) and 1363 cm^−1^ (34%). With the introduction of magnetic field (A (MF)), there were noticeable definitions and enhancements in some bands like 613, 671, 773, 1126, 1182, 1363, 1483, 1508, 1572 and 1651 cm^−1^. This outcome clearly points out the effect of magnetic polarisation of plasmon electrons at local sites between Fe_3_O_4_ and Au, thus increasing the electron density. This density enhances the vibrational response of bonds within R6G.

The same effect seen in Region A (Fig. 9(b)) was realised in Region B (Fig. 9(c)) and Region C (Fig. 9(d)), except for the relatively increased intensities. This can be attributed to the increasing concentrations of Au from regions A to C, as identified from XRD measurements. Overall, from the observation of all R6G magneto-Raman readings in Fig. 9, bands 613, 773, 1126, 1182, 1363, 1508, 1572 and 1651 cm^−1^ exhibited more distinct profiles compared to readings without a magnetic field, especially for 1508 cm^−1^ yielding approximately 133% intensity increase. This value was estimated using peak intensity averaging, $\:{I}_{avg}\left(v\right)=\frac{1}{N}{\sum\:}_{i=1}^{N}{I}_{i}\left(v\right)$, from multiple scanned data.

## Discussion

Results from image analysis, XRD and UV-Vis-NIR spectroscopy reveal a trend of higher concentrations of Fe at location A with a decreasing concentration through to point C. In comparison, Au concentrations across the three locations which were the opposite. From the thin film preparation process under magnetic field and spin coating, the relatively higher magnetic field in location A led to higher packing energy and concentrations of Fe (referring to the image analysis). This packing energy and high cluster density potentially led to increased pressures (laplace pressures) across the interfaces of the Pickering droplets. This could have led to the expulsion of some Au nanoparticles, reducing the concentration in that region. As for the other locations, B and C, the packing energies were lesser, initiating lesser dislodgment and displacement of Au nanoparticles.

**Fig. 10:**
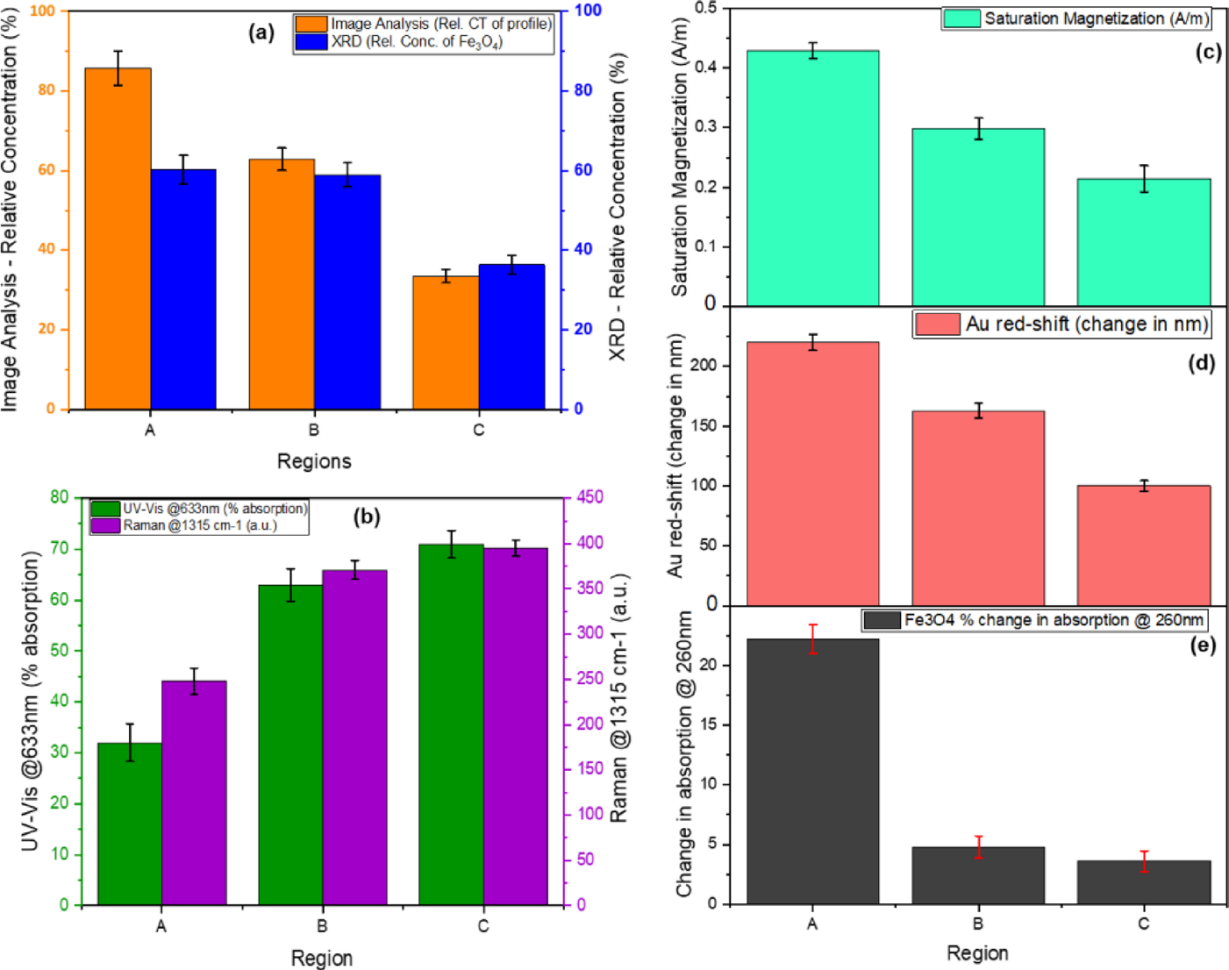
Statistical relationship of results across each region A, B & C; **(a)** Bar chart of the relative concentration of average Chain thickness (CT) from image analysed scan profile, along with the relative concentration of Fe_3_O_4_ nanoparticles within the combination of Fe_3_O_4_ and Au intensity readings estimated from XRD (Fe_3_O_4_ {311} & Au {111}) measurements. **(b)** This combo chart shows the effective relationship between patterned thin film UV-Vis-NIR plasmon absorption at 633 nm Raman probe laser wavelength and the R6G Raman intensity at 1315 cm^−1^ Raman shift. **(c)** Is the saturation of magnetisation, **(d)** is the percentage change in Fe_3_O_4_ reflectance at 260 nm upon the presence and absence of a magnetic field. **(e)** is the bathochromic shift (nm) of Au plasmon d without a magnetic field.

The relative comparison of the chain thickness (CT) via image analysis (Fig. 5) majorly consists of magnetically clustered Fe_3_O_4_ nanoparticles which is a relative percentage of nanoparticles in each of the scanned regions A, B and C. The relative percentage of Fe_3_O_4_ nanoparticles in its combination with Au was quantified from the XRD measurements using Fe_3_O_4_ (311) and Au (111) (Fig. 7); where for example, in region A, there was an average of 88% occupied by nanoparticles, of which 60% is Fe_3_O_4_ (Fig. 10a). This means the remaining 40% of Au nanoparticles exist either above or in-between the Fe_3_O_4_ nanoparticles. The low percentage of Au in this region agrees with the UV-Vis-NIR spectra in Fig. 6 (a)-line A. There are two distinct peaks in 610 nm and 802 nm associated with Au, showing a partial interaction with Fe_3_O_4_ (at the top surface), leading to minor bleaching of the Au nanoparticles. Meanwhile, for Au nanoparticles in between Fe_3_O_4_ nanoparticles, it potentially leads to the extreme bleaching of Au, resulting in a red-shifted absorption at 802 nm. This effect also contributes to the enhanced absorption of Fe_3_O_4_ at 260 nm.

As for region B, the relative concentrations of Fe_3_O_4_ and Au are 57% and 43%, respectively, due to sufficient chain gaps accounting for approximately 37% (Fig. 10a). The UV-Vis-NIR spectrum shows a broad absorption band at 630 nm for Au (Fig. 7b). This occurred likewise for Region C, with more absorption at 630 nm than Region B for Au (Fig. 7c). This makes sense because the relative concentration of Au is approximately 30% more than that of Fe_3_O_4_ (Fig. 10a).

As earlier highlighted regarding the cause for the redshift of Au plasmon resonance, which shows a highly electron-deficient Au with its electrons diffusing towards multiple interfacial contacts with Fe_3_O_4_. Series of investigations carried out on relative concentrations of Fe_3_O_4_ and Au dimer system, especially for Fe_3_O_4_-Au core-shell nanoparticles, revealed that the smaller the concentration of Au the more redshifted and lower intensified the plasmon resonance peak becomes^87–89^. Such configurations were expected, with more Fe_3_O_4_ nanoparticles/atoms than Au nanoparticles, which led to the realisation of such spectra as shown across all three regions (Fig. 8). This is deemed to be consistent with an investigation carried out by George and Co., when comparing spherical and rod-like Fe_3_O_4_ dimer structures with Au nanoparticles^90^. They indicated that faster dynamics of charge mobilisation was not only responsible, but also fast trapping of hot electrons from Au at the numerous defect sites at the interface. In addition, an increase in Au concentration per unit concentration of iron oxide leads to a blueshifted plasmon resonance absorption band from 690 nm, followed by a decrease in absorption intensity for Fe_3_O_4_ at 400 nm.

The introduction of a magnetic field strongly influenced the photoabsorption of components within the thin film fabrication. The local magnetic moment of Fe_3_O_4_, in combination with the nanoparticle concentration, strongly influenced the saturation of magnetisation as discovered from VSM measurements (magnetic hysteresis curve output – Fig. 6). The magnetic saturation across the three regions is related to both the change in absorption intensities of Fe_3_O_4_ and plasmon bathochromic shift values of Au, see Fig. 10(c-e). The effect of the magnetic field on the UV-Vis-NIR measurements of Fe_3_O_4_ nanoparticles at ~ 260 nm absorption band is an interesting phenomenon. It is hypothesised that the polarisation of the electrons by the magnetic field limited the oscillation behaviour of the nanoparticles, thereby maintaining the charged electrons at the interfaces of the nanoparticles^40^. This behaviour is significant in region A, with an absorption decrement of 22%, where there is a higher density of contacting Fe_3_O_4_ nanoparticle surfaces than in regions B and C (Fig. 10d). It can be deduced from the plots (Fig. 10) that Fe_3_O_4_ nanoparticle concentration influences the density of magnetic moment polarisation, which in turn influences the polarisation of plasmon electrons from Au nanoparticles. The concentration ratio between both materials also affects the density of electrons that could be polarised and thus realised from the degree of red-shifts of Au plasmon peaks (Fig. 10e).

In conclusion, the magnetic field generally shifts the dielectric properties of all samples towards more favorable plasmonic conditions at 633 nm, with Sample B showing the most ideal characteristics for SERS enhancement under a magnetic field. Sample C undergoes a significant transformation from non-plasmonic to potentially plasmonic behavior with the application of a magnetic field.

The magneto-SERS system clearly showed the effect of locally densified magnetically polarised plasmon electrons on the sensitivity of probed molecular species. The average effective increase of Raman signal intensities coincides with the increase in Au concentrations relative to the scanned regions from XRD measurements. This is also significantly relatable to the absorbance intensity of the patterned thin film at the Raman probe laser wavelength of 633 nm. By taking absorption intensity to be 100% minus percentage reflectance, the values for each scanned region were compared with the intensities of one of the most sensitive R6G Raman shift bands in 1315 cm^−1^ (see Fig. 10b). Taking the readings from non-magnetised spectra in Fig. 9, the respective R6G Raman shift intensities of 1315 cm^−1^ relatively identifies with the increasing photoabsorption in each region of the patterned thin film material at 633 nm.

Apart from the obvious increased intensities of some Raman shift bands under magnetic field (MF) stimulation across the regions of interest, the overall outlook between the presence and absence of MF is not much different. However, the bands of R6G under the influence of MF like 1126, 1182, 1448 and 1572 cm^−1^ were highly distinct and detectable. This paradigm could be useful in fine-tuning vague Raman spectra of biomolecular specimens with complex molecular frameworks.

## Conclusion

In this work, a fabricated Fe_3_O_4_-Au composite system was tested for its effectiveness in biosensing applications like SERS. Especially with the agglomerate ordering of the composite nanoparticles by magnetic stimulation of magnetic responsive iron oxide (Fe3O4). This densified multistructural agglomerate template exhibited varying plasmonic charge dynamics from interfacing Fe_3_O_4_ and Au nanoparticles set-up to enhance the vibrational signals of analyte molecules, like Rhodamine 6G (R6G). It was realized that the concentration density and ratio between Fe_3_O_4_ and Au within the thin film cast influenced the optoelectric response of the system when irradiated with light. This behaviour varied even more under the presence of a magnetic field where theoretically the local magnetic moment of polarization of Fe polarons effectively influenced the plasmon electrons of Au nanoparticles at the interface between Fe_3_O_4_ and Au. This key difference under the presence and absence of magnetic field was noticed under UV-Vis-NIR and SERS measurements, particularly in the magneto-SERS measurements of R6G, where the vibrational band signals were higher and more distinguishable.

Overall, the design of thin-film patterned heterostructures, concentration ratios, and configurations allowed for local field energy and intensity tunability of vibrational signals of biomolecular species under SERS and potentially SEIRA measurements. Based on the practical effect of external magnetic fields in optical diagnostics from this study, it is suspected that implementing permanent magnets beneath the substrate may not be the optimal setup going forward; therefore, several configurations using uniform magnetic fields from electromagnets directed parallel or perpendicular to the surface of the substrate might yield a more deterministic outcome.

## Supplementary Information

Below is the link to the electronic supplementary material.

Supplementary Material 1

## Data Availability

All data generated or analysed during this study are included in this published article [and its supplementary information files].
