# Supplementary material for: The optoelectric tunability effect of structurally patterned Fe3O4-Au assembly on Rhodamine 6G signals under Magneto-SERS measurements
Source: Sci Rep. 2025 Oct 28;15:37643. doi: 10.1038/s41598-025-21536-y (PMC12568977; doi:10.1038/s41598-025-21536-y)
Supplement: Supplementary file 1 — Supplementary Material 1 [file 41598_2025_21536_MOESM1_ESM.docx]

Supporting Information

Title: **The Optoelectric Tunability effect of Structurally Patterned Fe_3_O_4_-Au assembly on Rhodamine 6G Signals under Magneto-SERS measurements**

Authors: *Paul Okpozo^1,*^, Jordan C. Kelly^2^, Jennifer A. Aitken^2^, John Viator^3^, Ketan Pancholi^1,*^*

^*^ E-mail: [p.okpozo@rgu.ac.uk](mailto:p.okpozo@rgu.ac.uk), [p.okpozo@outlook.com](mailto:p.okpozo@outlook.com), k.pancholi2@rgu.ac.uk

^1^School of Engineering, Robert Gordon University, Aberdeen, AB10 7GJ, UK.

^2^Department of Chemistry and Biochemistry, Duquesne University, 600 Forbes Avenue, Pittsburgh, PA 15282, USA.

^3^Biomedical Engineering Department, Duquesne University, 309 Libermann Hall

Pittsburgh, PA 15282, USA.

1. **Electron Micrograph Samples of Scanned Regions A, B and C**


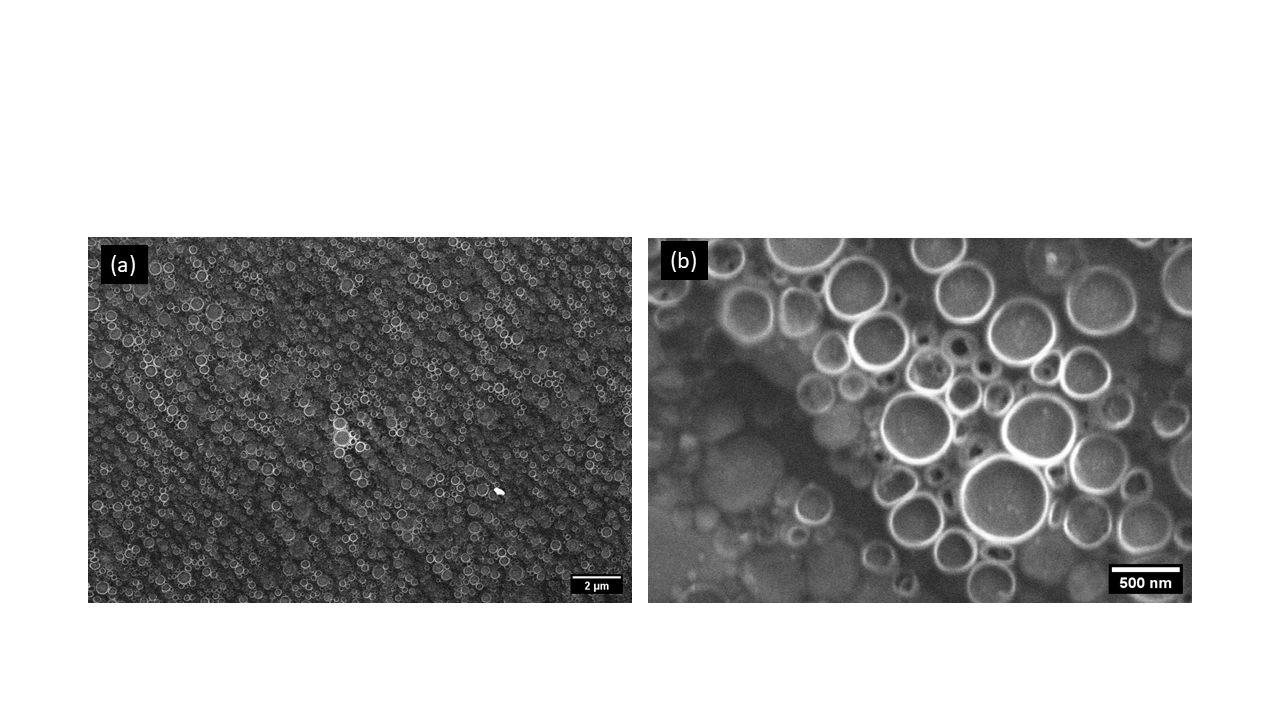


Figure S-1: SEM of scanned region A.


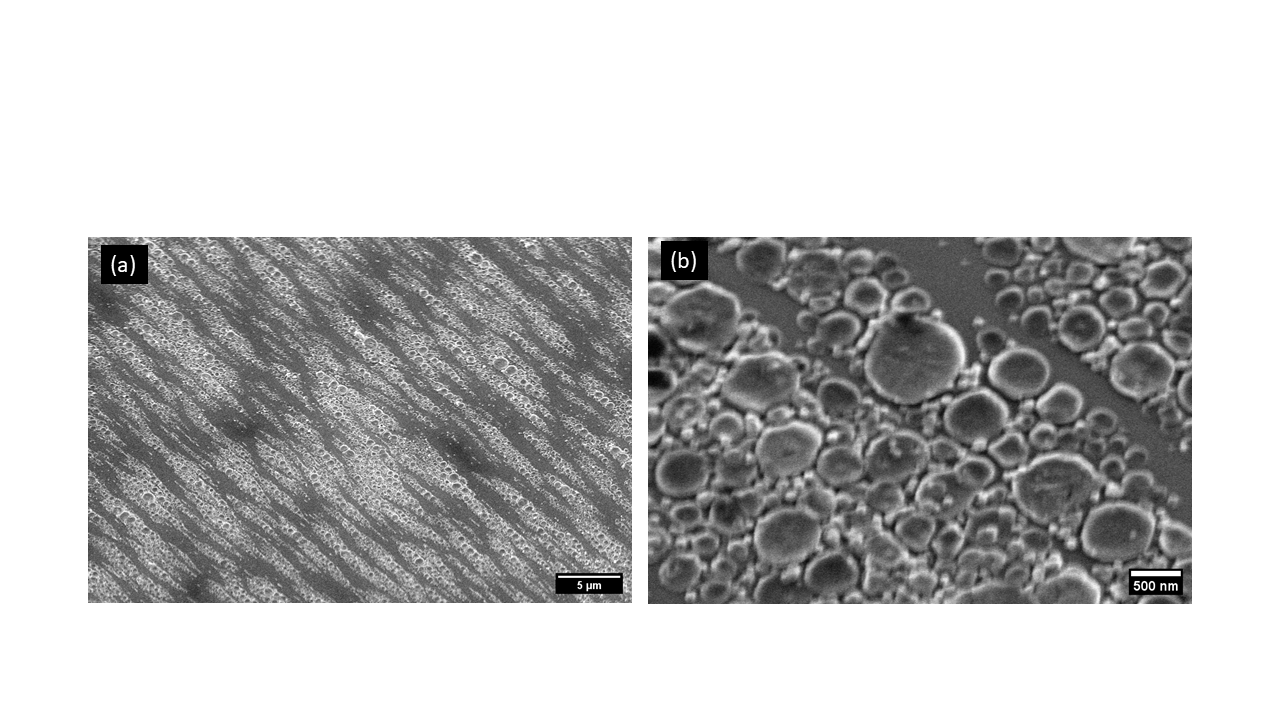


Figure S-2: SEM of scanned region B.


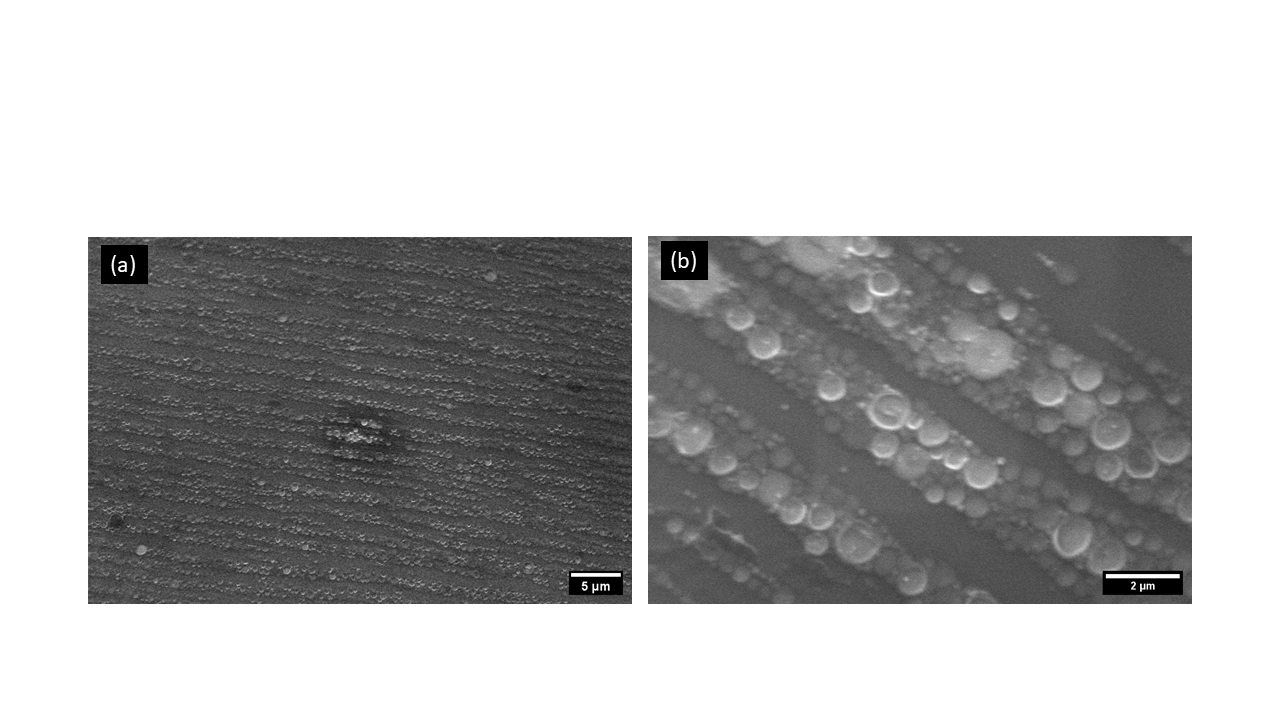


Figure S-3: SEM of scanned region C.

1. **Image Processing Technique**
   1. **Algorithm for Measuring the Chain thickness and gap Profile scan**


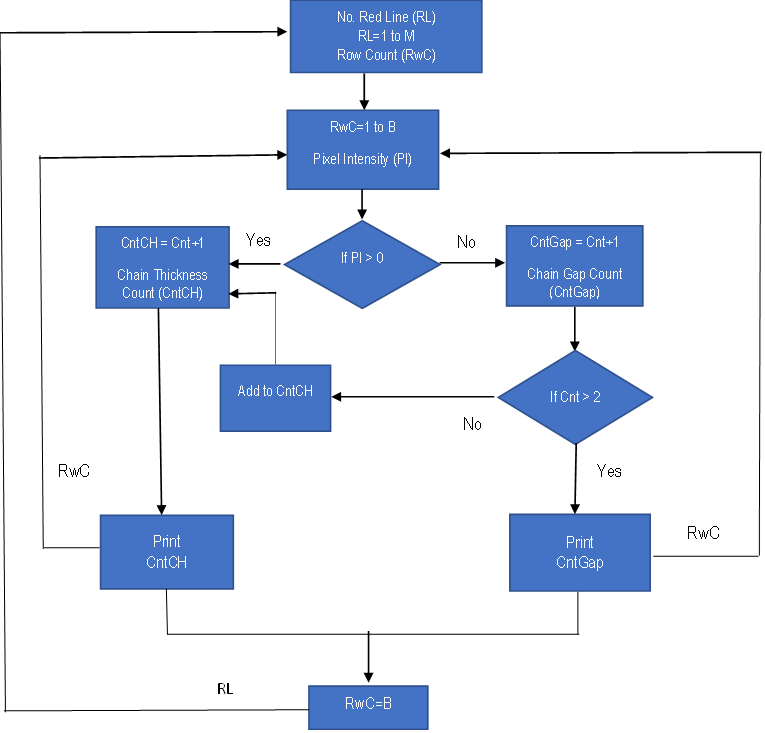


- 1. **Microsoft Visual Basic Code for Chain thickness and gap Profile scan**

Sub CL-CT_Scan()

Dim RL, M, rw, CntCH, CntGap, K, L, CL1, CL2, RwC, W As Integer

'This is for estimating the chain thickness and gap from image scan

‘i is counter for number of column/lines to be scanned, M = Number of ‘scanned ‘lines, CntCH = Chain thickness pixel counts, CntGap = Chain ‘gap pixel ‘counts, K, CL2 = variable associated with CntCH, L, CL1 = ‘variable ‘associated with CntGap, d = pixel count (rows - W).

M = Sheet3.Cells(30, 25).Value 'number of columns to be scanned

For RL = 1 To M

CntCH = 0

CntGap = 0

K = 1

L = 1

W = Sheet3.Cells(30, RL * 2 + 24).Value 'number of rows per column

For RwC = 2 To W Step 1

If Sheet3.Cells(RwC, 2 * RL).Value > 0 Then

If CntCH = 0 Then

K = K + 1

CL2 = K

Else

K = CL2

End If

CntCH = CntCH + 1

Sheet3.Cells(K + 31, RL * 2 + 24) = CntCH

CntGap = 0

Else

If CntGap = 0 Then

L = L + 1

CL1 = L

Else

L = CL1

End If

CntGap = CntGap + 1

Sheet3.Cells(L + 31, RL * 2 + 25) = CntGap

CntCh = 0

End If

Next RwC

Next RL

End Sub

1. **Supplementary info for Raman laser power intensity test**

The Raman spectra of crystalline silicon wafer with the long distinct peak at 520 cm^-1^ is the first order Raman active phonon located at the Brillouin-zone centre. This first order Raman (stokes) spectrum arises from the creation of the triply degenerate, long wavelength transverse optical phonon (TO) [47, 48]. The insert in **Figure S-4 (a)** is the intensity of the 520 cm^-1^ plotted against incident % power. It shows that with increase in power leads to increase in overall spectrum intensity. The next case involves testing the substrate coated with the thin film patterned structure under 0.1% incident laser power. **Figure S-4 (b)** shows the spectra at regions A, B and C within the substrate. The insert shows the 520 cm^-1^ band peak plotted against the selected regions. It can be observed that the peak intensity of region C from the insert plot is approximately 35 times more than the bare silicon wafer probed with the same laser power (**Figure S-4 a**). This means the nanoparticle patterned film local plasmon intensity generated a similar probe laser intensity of 10% (Figure S-4 a – insert).


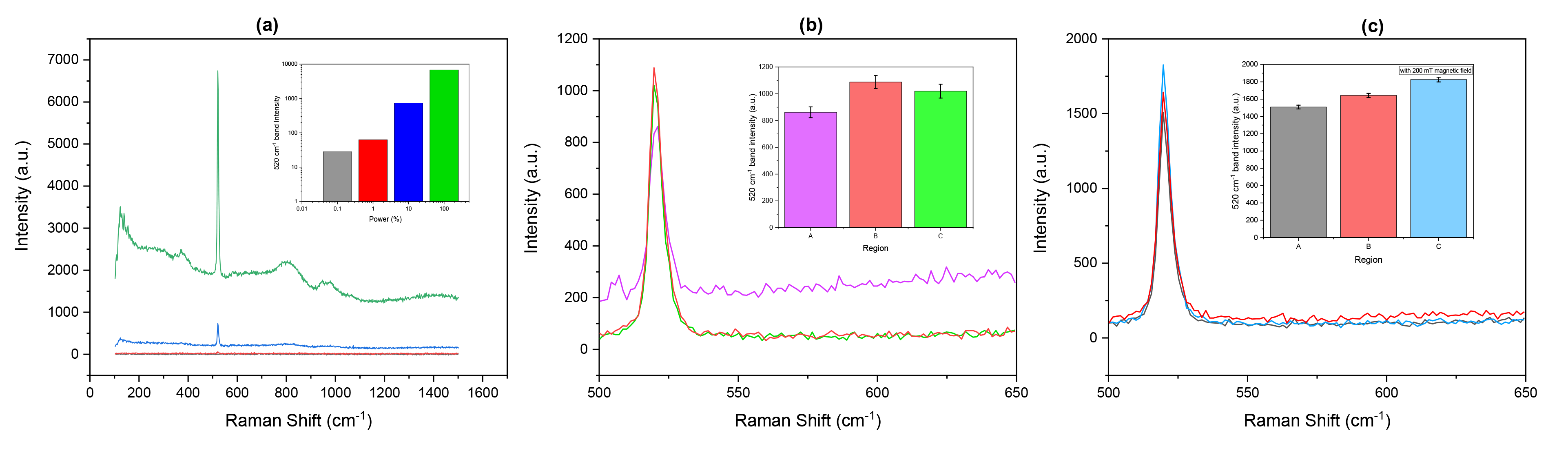


**Figure S-4**: Raman spectroscopy test of silicon wafer with 520 cm^-1^ as reference point (a) under various laser power (b) with nanoparticle coating (c) with nanoparticle coating and 200 mT magnetic field.

In another test, a magnet was placed underneath the substrate, parallel to the direction of the probing beam on the sample. The average magnetic field across the scanned areas was tested and measured to be approximately 200 mT. **Figure S-4 (c)** represents the Raman spectra of the sample under the influence of magnetic field. The insert shows the 520 cm^-1^ band peak plotted against the regions of interest of the sample. The intensity of signal rose by 78% beyond the intensity of the signal without a magnet (for region C). This is a positive indication that the sample is generally magneto-optically responsive.

## **References**

1. Huong, P.V., Materials and Interfaces characterisation by micro-raman spectroscopy. *Journal De Physique IV,* 1(3), *(1991)*.
2. Jeong, J., Chung, S.G., Nishino, S. Raman Scattering investigation of polycrystalline 3C-SiC Film deposited on Sio2 by using APCVD with Hexamethyldisilane. *Journal of Korean Physical Society, 52(1),* pp. 43-47 *(2008)*.
